# When good bacteria behave badly: a case report of *Bacillus clausii* sepsis in an immunocompetant adult

**DOI:** 10.1099/acmi.0.000097

**Published:** 2020-02-03

**Authors:** Isabella Princess, T. Natarajan, Siddhartha Ghosh

**Affiliations:** ^1^​ Department of Microbiology, Apollo Speciality Hospitals, Vanagaram, Chennai – 600095, India; ^2^​ Department of Neurosciences, Apollo Speciality Hospitals, Vanagaram, Chennai – 600095, India

**Keywords:** *Bacillus clausii*, probiotics, blood culture, sepsis

## Abstract

Reports of unusual microorganisms causing human infections are on the rise due to transitions in epidemiological trends. Commensal/normal flora which are otherwise termed as ‘good bacteria’ are now causing infections in different group of patients, mostly immunocompromised individuals. Various host and environmental factors play a pivotal role in microbial transmigration from their normal habitat into the blood and other body sites. We report one such ‘good bacterium’ associated with sepsis in a patient who was given the same bacterium in the form of probiotics.

## Introduction

Probiotics are live microorganisms which have the ability to colonize the human gut and provide health benefits [1]. Organisms commonly used as probiotics include *Lactobacillus *, *Bifidobacterium *, *Bacillus *, *Enterococcus *, *Escherichia *, *Streptococcus* or fungi such as *Saccharomyces boulardi* [2, 3]. The beneficial effects of probiotics documented in the literature include: synthesis of vitamins, prevention of colonization of pathogenic bacteria, antagonization of other bacteria by secreting bacteriocins and other antibacterial substances, and stimulation of secretory IgA antibodies which antagonize other pathogens [3]. These benefits make them a potential option in treating antibiotic-associated colitis, critically ill and surgical patients, and diarrhoea in children and adults.

Although the beneficial effects of probiotics are well publicized, the risk and drawbacks due to the same is almost always neglected. There have been reports of various probiotic bacteria such as *Bacillus subtilis* causing sepsis and a few recent reports from India and the Philippines have surfaced on *Bacillus clausii* sepsis in immunocompromised individuals and in neonates [4]. We encountered a diabetic but otherwise normal adult developing sepsis with *B. clausii *, the source of which was traced to be the same organism given to her in the form of probiotics. To the best of our knowledge, this is the first report of *B. clausii* causing sepsis in an immunocompetent adult.

## Case report

Our patient is a middle-aged type II diabetic, not a known hypertensive, with no history of any other chronic illness and no other significant past or family history. The patient presented to the emergency department with acute-onset frontal headache of 1 day duration. A diagnosis of cerebral vein thrombosis with right parietal intraparenchymal bleeding with oedema and midline shift was made. An emergency decompressive craniotomy was done and the patient was gradually weaned off the ventilator, the tracheostomy tube was decannulated, she was mobilized and her Glasgow Coma Scale (GCS) improved to 15/15.

After 26 days, the patient underwent autologous and mesh cranioplasty. She then developed subgaleal haematoma 7 days after the cranioplasty for which re-exploration and evacuation of the haematoma was performed. Recurrent subgaleal and extradural haematoma with midline shift developed with a drop in GCS to 5/15. The patient underwent emergency re-exploration and evacuation of the haematoma with bone flap removal with significant blood loss which was corrected with transfusion of 6 units of blood and blood products. The patient was being continuously monitored in the Neurosurgical intensive care unit and treated with appropriate medication after four surgical procedures.

**Fig. 1.**
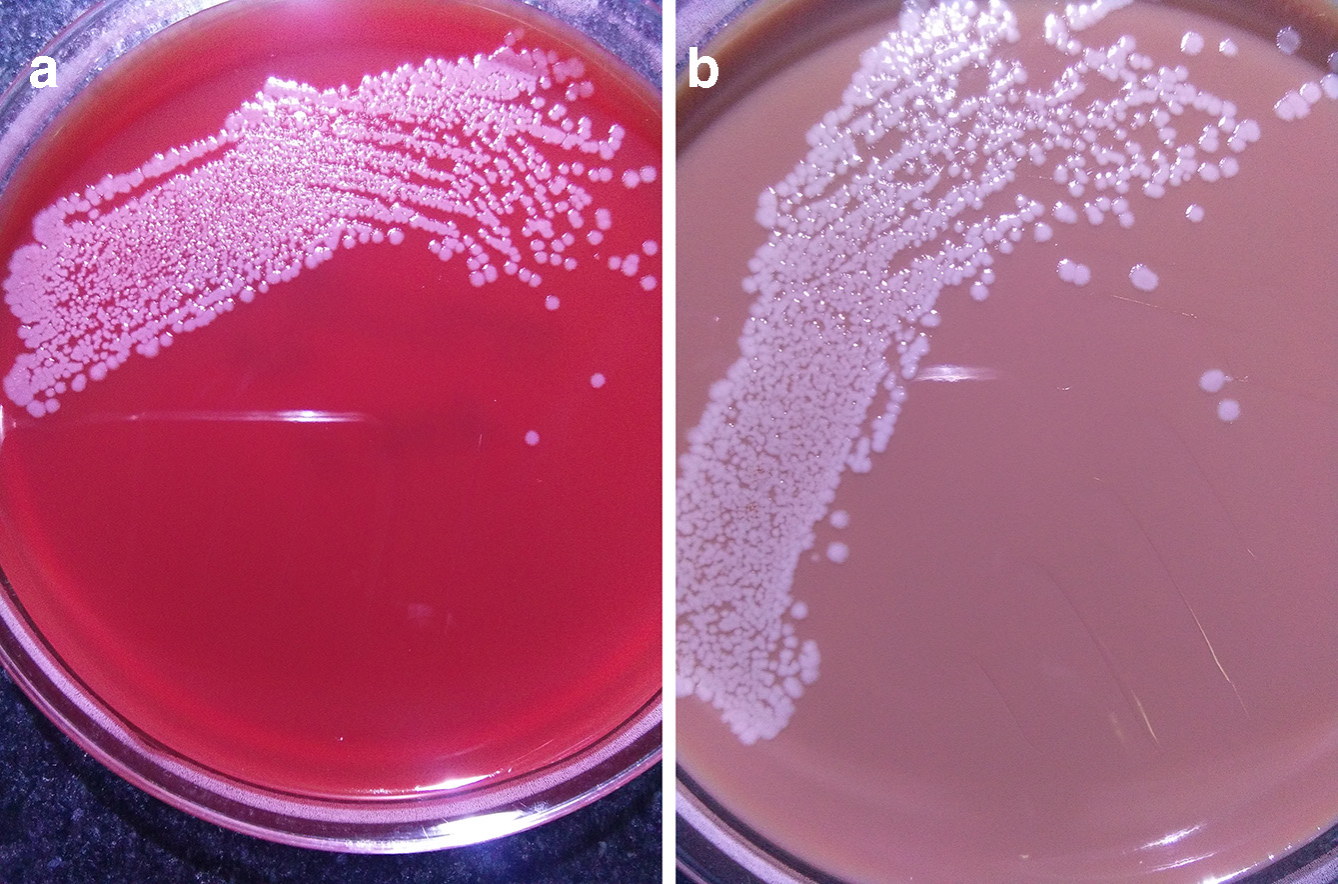
Culture characteristics of the *Bacillus clausii* isolate from blood on (a) blood agar (colony variants) and (b) chocolate agar.

**Fig. 2.**
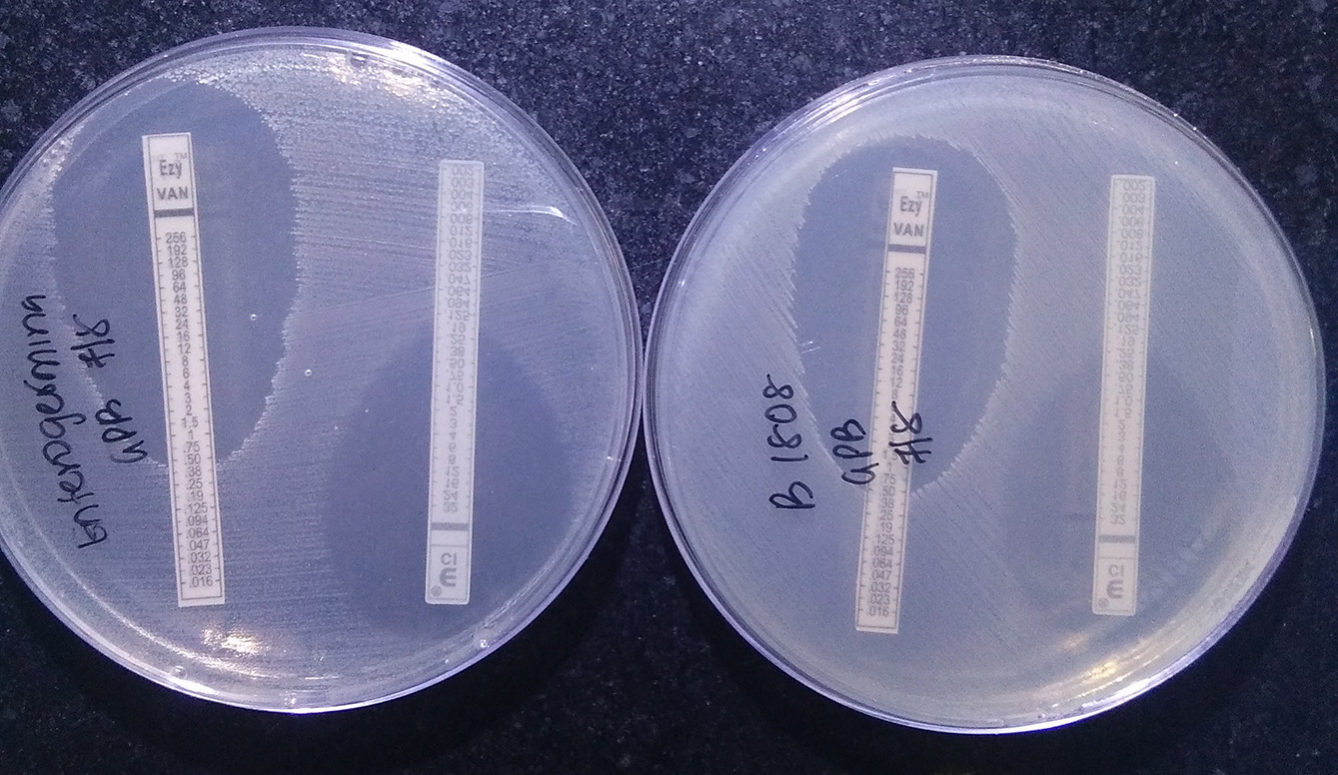
Antibiotic susceptibility testing of the clinical isolate (right) and probiotic strain (left)

On the third post-operative day of the last surgery, a tracheal secretion grew *Klebsiella pneumoniae* which was multidrug-resistant. Drain fluid and pus taken from the surgical site also grew multidrug-resistant *K. pneumoniae* which was successfully eliminated with antimicrobial therapy. The patient was treated with appropriate antibiotics and with the probiotic Enterogermina (* B. clausii* spore suspension of 2 billion/5 ml, Strains: O/C, N/R, SIN and T) [5] due to loose stools on broad-spectrum antibiotics. After about 10 days, the patient developed fever after which two sets of blood cultures were drawn from two different sites (peripheral venipuncture). Procalcitonin as determined by semi-quantitative immunochromatography was >0.5 to ≤2 ng ml^−1^. Two aerobic blood culture bottles grew Gram-positive bacilli within 48 h which were isolated in pure culture and identified as *B. clausii* by a matrix assisted laser desorption ionization time of flight (MALDI-TOF; bioMeriéux) assay (Fig. 1). Growth was observed 11 days after initiation of the probiotic containing spores of *B. clausii *. Phenotypic drug susceptibility testing for determination of MIC using an E test was performed using Clinical Laboratory Standards Institute (CLSI) M45 guidelines [6]. The isolate was susceptible to ciprofloxacin (MIC: 0.38 µg ml^−1^) and vancomycin (MIC: 0.5 µg ml^−1^) but resistant to penicillin (MIC: 32 µg ml^−1^). All other recommended antibiotics were tested using Kirby Bauer disc diffusion but are not reported because there are no interpretative CLSI guidelines. In order to determine the origin of the blood isolate, the probiotic was subjected to identification following aerobic culture. We were able to isolate and identify the organism as *B. clausii* using MALDI-TOF with the susceptibility pattern of the probiotic strain perfectly matching the blood isolate (Fig. 2). These phenotypically correlative findings between the clinical isolate and the probiotic stain established that the probable source of *B. clausii* in blood to be from the probiotic. The patient was treated with teicoplanin, which showed an MIC of 0.094 µg ml^−1^, although it could not be reported as susceptible due to the unavailability of interpretative guidelines. The patient responded well to antibiotic therapy and became afebrile with very good resolution of sepsis within 48 h of teicoplanin initiation. Repeat blood cultures drawn 2 weeks after initial blood cultures showed no growth, thereby signifying adequate response to antibiotic therapy.

## Discussion

The usefulness and disadvantages of using *B. clausii* spores as probiotics in critically ill patients is discussed here. It is noteworthy that *B. clausii* in our patient is definitely a rare occurrence compared to previous reports of *B. clausii* sepsis. Our patient did not have any underlying immunocompromising condition such as malignancy, corticosteroid therapy, transplantation or immunodeficiency syndromes. This observation is contrary to previously published reports of *B. clausii* associated with sepsis in individuals with one of the above listed immunocompromising conditions [7, 8]. Therefore it is noteworthy that *B. clausii* used as probiotics can transmigrate from the gut and enter the bloodstream of an individual irrespective of his/her underlying immunocompromised state.

Another major lesson from our patient is the need to send at least two sets of blood cultures in clinically suspected sepsis patients [9]. Additional blood culture bottles increase the sensitivity and yield of microorganisms causing sepsis [10]. It is common practice to ignore Gram-positive bacilli as a contaminant from one bottle of blood because *Bacillus* is one of the most common contaminants [11]. In our patient, the organism was isolated from two (aerobic) bottles out of four (two aerobic and two anaerobic) bottles of blood sent for culture. In order to establish the pathogen it is convenient to collect an adequate quantity of blood, and convincing evidence would be isolation of the organism of questionable significance from more than one blood culture bottle.

In previously published reports, vancomycin, a glycopeptide group antibiotic, has been used in treating patients with *B. clausii* sepsis. Our patient was treated with another glycopeptide, teicoplanin, and successful elimination of the bacterium from blood was achieved. Since interpretative guidelines for teicoplanin are not given by the CLSI, the same could not be interpreted from the patient's isolate. However, following successful treatment in our patient, it can be postulated that teicoplanin is an effective alternative in treating sepsis due to *B. clausii *. A major challenge in treating *B. clausii* sepsis is the limited therapeutic options. This is due to the fact that *B. clausii* is known to carry multiple drug-resistant genes [12], and could explain treatment failure in patients from previously published reports. Initiation of an appropriate antibiotic as well as adequate dosage was another positive aspect of our case which resulted in better patient outcome. Therefore, thorough knowledge of resistance patterns is mandatory when dealing with infections caused by exotic/unusual organisms.

Good bacteria can turn bad, good bacteria can carry drug-resistant genes [8], and good bacteria can be recalcitrant to therapy and require high-end antibiotic therapy. Bacterial strains used as probiotics can become virulent and establish themselves as pathogens. The mechanism of virulence remains questionable especially in normal individuals, warranting further research on this issue.

### Conclusion

*B. clausii* may be safe as a probiotic but its use in immunocompromised and chronically ill patients is questionable. This therapeutic dilemma is due to its probable ability to transmigrate from the gut into the bloodstream and cause sepsis, as experienced by our patient. There are no previous reports of *B. clausii* sepsis among adults with no known underlying chronic disease or immunocompromising condition. This case report can thus be taken as a wake-up call for making judicious use of this probiotic even in normal individuals. We conclude by stating that use of probiotics containing *B. clausii* spores in critically ill patients is not always beneficial; rather its use should be used with caution.
